# Metformin and insulin treatment prevent placental telomere attrition in boys exposed to maternal diabetes

**DOI:** 10.1371/journal.pone.0208533

**Published:** 2018-12-11

**Authors:** Isabel Garcia-Martin, Richard J. A. Penketh, Anna B. Janssen, Rhiannon E. Jones, Julia Grimstead, Duncan M. Baird, Rosalind M. John

**Affiliations:** 1 Division of Biomedicine, Cardiff School of Biosciences, Cardiff University, Cardiff, Wales, United Kingdom; 2 Department of Obstetrics and Gynaecology, University Hospital Wales, Cardiff, Wales, United Kingdom; 3 Division of Cancer and Genetics, Cardiff School of Medicine, Cardiff University, Cardiff, Wales, United Kingdom; University of Missouri Columbia, UNITED STATES

## Abstract

Shortened leukocyte and placental telomeres associated with gestational diabetes mellitus (GDM) suggest this exposure triggers telomere attrition contributing to adverse outcomes. We applied high resolution Single Telomere Length Analysis (STELA) to placenta from GDM pregnancies with different treatment pathways to determine their effectiveness at preventing telomere attrition. Differences in telomere length between control (N = 69), GDM lifestyle intervention (n = 14) and GDM treated with metformin and/or insulin (n = 17) was tested by Analysis of Covariance (ANCOVA) followed by group comparisons using Fisher’s least significant difference. For male placenta only, there were differences in mean telomere length (F(2,54) = 4.98, *P* = 0.01) and percentage of telomeres under 5 kb (F(2,54) = 4.65, *P* = 0.01). Telomeres were shorter in the GDM lifestyle intervention group compared to both controls (*P* = 0.02) and medically treated pregnancies (*P* = 0.003). There were more telomeres under 5 kb in the GDM lifestyle intervention group compared to the other two groups (*P* = 0.03 and *P* = 0.004). Although further work is necessary, we suggest that early adoption of targeted medical treatment of GDM pregnancies where the fetus is known to be male may be an effective strategy for ameliorating adverse outcomes for children.

## Introduction

Gestational diabetes mellitus (GDM) can be defined as any degree of glucose intolerance with onset or first recognition during pregnancy [1]. One in seven births is affected by gestational diabetes (GDM) and these numbers are expected to increase in the years ahead, as reported by the International Diabetes Federation. During a normal pregnancy, the maternal system develops insulin resistance, increasing progressively towards term, to ensure nutrients are channelled to the developing foetus. GDM is thought to arise when peripheral insulin resistance exceeds an appropriate threshold, resulting in hyperglycaemia. The development of GDM involves both genetic and lifestyle factors, with overweight and obese women at highest risk [2]. While GDM can be controlled by diet or drugs, uncontrolled GDM increases the risk of delivering a large for gestational age baby (birth weight above the 90th percentile) and delivery by Caesarean section [3]. Women diagnosed with GDM are more likely to experience pregnancy related depression [4] and seven times more likely to develop type 2 diabetes mellitus (T2DM) later in life [5]. Their children are less likely to reach timely developmental milestones [6] and more likely to be overweight and with a six-fold increased risk of T2DM [7, 8]. Given the increasing prevalence of maternal obesity worldwide, understanding the consequences of GDM has even greater significance.

A number of studies report that individuals with T2DM have shorter telomeres [9]. Telomeres, which consist of the repeated DNA sequence TTAGGG, acts as caps at the ends of chromosomes [10] to maintain genomic stability [11]. Telomeres shorten with progressive cell division, due to the inability of conventional DNA polymerase to complete the replication of chromosome ends. While the ribonucleoprotein enzyme telomerase adds telomere repeats to mitigate telomere erosion, the majority of somatic cells lack significant telomerase activity [12, 13] consequently telomeres progressively shorten as cells divide and as individuals age. The presence of shortened telomeres in T2DM patients suggests the possibility that diabetes contributes to telomere erosion. Conversely, individuals may start life with shorter than normal telomeres that then play a role in the disease progression of diabetes [14], contributing to impaired glucose management and insulin secretion [15]. The latter possibility is indicated by the observation that diabetes during pregnancy, is linked to shortened telomere length in blood leukocytes from exposed children [16, 17], although not all studies report such a link [18–20]. If individuals exposed *in utero* to GDM start life with shorter telomeres, this may explain why they are at higher risk of adverse outcomes such as T2DM later in life. However, fetal tissues are not easily accessible for measuring properties of DNA and there are considerable issues with the use of heterogeneous white blood cell samples [21]. In contrast, the placenta is a fetally-derived tissue exposed to the same environment as the foetus with a limited cellular heterogeneity [22], in which telomere length can easily be determined. An association between GDM and shorter telomere length in the placenta has been reported using the fluorescence *in situ* hybridization (FISH) assay on 16 samples [23]. In this study, shortened telomeres were defined by a weak signal intensity, and further analysis revealed significantly lower telomerase expression in these same samples [20]. Despite the clinical importance of GDM, few studies have examined placental telomeres in relation to GDM, and none have applied higher resolution assays.

We recently applied Single Telomere Length Analysis (STELA) to placental telomere analysis [24]. STELA is a single-molecule PCR based telomere length analysis technology that determined the telomere length profiles from single chromosome ends [25]. This high-resolution technique provides information on individual telomere length which can reveal additional information such as the remarkable heterogeneity present in term placenta [24]. In conjunction with University Hospital of Wales and Royal Gwent Hospital, we engaged in a study to investigate placental telomere from term pregnancies in Welsh women. From these populations, we collected placenta from uncomplicated and GDM pregnancies making a note of their treatment pathways, and applied STELA.

## Materials and methods

### Participant recruitment

Study participants (N = 100) were recruited and consented prior to delivery at University Hospital Wales and Royal Gwent Hospital as described previously [26]. Ethical approval for the human study “Determining the expression levels of a set of imprinted genes in the term placenta from both normal and atypical pregnancies” was obtained via South East Wales Research Ethics Committee Panel B in 2010, REC number 10/WSE02/10. Sponsor: NHS–Cardiff and Vale ULHB. Ethical approval for the human study “The Grown in Wales Study: Developing a placentomic tool for characterising atypical pregnancies and predicting outcomes” was obtained via Wales Research Ethics Committee 2 2015, REC number 15/WA/0004. Research was carried in accordance with the principles of the Declaration of Helsinki as revised in 2008.

### Placental biopsies

Term placenta (37–42 weeks) were collected by trained research midwives within two hours of an elective caesarean section from singleton pregnancies. Unless otherwise stated, chorionic villous samples were taken 1 cm below the surface from the maternal side of the placenta at 5 different sites midway between the cord insertion and the lateral edge. Samples were washed three times in phosphate buffered saline and stored in RNAlater at -80°C until needed.

### Subjects

In Cardiff and Vale, high risk women (defined by a BMI over 30, prior history of gestational diabetes or macrosomic infant, family history of diabetes and Asian, African-Caribbean or Middle Eastern origins) are routinely offered glucose tolerance testing. Women with either a fasting plasma glucose level of 5.6 mmol/l or above, or a 2-hour plasma glucose level of 7.8 mmol/l or above, are initially offered dietary advice and recommended to undertake regular exercise with a common recommendation being at least 150 minutes (2 hours and 30 minutes) of moderate exercise, such as brisk walking or swimming, per week. If blood sugar levels remain elevated, women are prescribed medication which is usually metformin but can involve insulin injections for women who cannot take metformin or whose blood sugar is dangerously high. In this study 31 women with GDM were identified with a note being made of treatment pathways and 69 women were identified who had undergone GTT with no evidence of diabetes (**Table 1**).

**Table 1:** Maternal, birth and metabolic characteristics. Mean (SD) or number (%) is shown. Note: due to missing values, some numbers do not add up to 100%. *P* values were assessed using Student’s t test, Mann–Whitney test or χ^2.^test.

Study participants	GDM group	Control group	*P* VALUE
	N = 31	N = 69	
**Caucasian**	26 (84%)	62 (90%)	0.08
**Parity: Primiparous**	3 (9.7%)	7 (10.1%)	0.88
**Parity: Multiparous**	17 (54.8%)	51 (74%)	
**Maternal age**	33 (3.9)	31 (5.8)	0.20
**Maternal BMI**	33 (7.0)	31 (6.3)	0.26
**Weight gain**	10.9 (8.9)	13.3 (8.3)	0.35
**Elective C-section**	31 (100%)	69 (100%)	NA
**Smoking**	1 (3.2%)	8 (11.6%)	0.23
**Lowest income**	2 (6.5%)	9 (13%)	0.76
**Fasting plasma glucose (mmol/l)**	5.6(1.2)	4.55(0.5)	**<0.001**
**2 hours glucose (mmol/l)**	8.7(2.1)	5.46(1.3)	**<0.001**
**Birth weight (g)**	3714 (433.6)	3734 (562.0)	0.86
**Gestational age (weeks)**	38 (0.6)	39 (0.7)	**0.007**
**Placental weight (g)**	701 (131.8)	723 (133.4)	0.43
**Infant gender: Male**	20 (64.5%)	38 (55.1%)	0.37
**Infant gender: Female**	11 (35.5%)	31 (44.9%)	

### Single Telomere Length Analysis (STELA)

STELA was performed as described previously [27]. Briefly, genomic DNA for each sample was amplified in multiple reactions (usually six reactions per sample) using telorette2, teltail and telomere specific primer XpYpE2. Amplified telomeric DNA fragments were then resolved by 0.5% agarose gel electrophoresis, detected by Southern hybridisation using a TTAGGG repeat probe α-33P dCTP labelled (Perkin Elmer) before visualisation using a Typhoon FLA 9500 phosphoimager (GE Healthcare Life Sciences). Telomere length distributions were determined using the ImageQuant software and descriptive statistics generated.

### Statistics

All statistical analysis was performed using SPSS 23.0 for Windows. Data are expressed as means with standard deviation, or as numbers (%). Differences between groups were assessed using Student’s t test or Mann–Whitney U-test. χ^2^ test was used for categorical data. Relationships between the main dependent variable and other variables was analysed by simple linear regression, or by Hierarchical regression allowing the entry of multiple independent variables if significant at *P* < 0.05. Differences in telomere length between the control group, the GDM lifestyle intervention group and the GDM mothers treated with metformin and/or insulin were tested by Analysis of Covariance (ANCOVA). ANCOVA was conducted to adjust for maternal ethnicity and *P* ≤ 0.05 was considered statistically significant. This was followed by group comparisons using Fisher’s least significant difference (LSD) method.

## Results

### Maternal, birth and metabolic characteristics of the study participants

GDM mothers (n = 31) and control mothers (n = 69) were predominantly Caucasian, consistent with the local population (Table 1). As ethnicity [28], maternal age [29] and obesity [30] are all risk factors for GDM, controls were matched for these characteristics. Other than fasting plasma glucose and 2 hours plasma glucose levels, only gestational age was significantly different between the groups (*P* = 0.007) with GDM mothers, on average, giving birth one week earlier than controls.

### Association between telomere length and potential confounders

We have previously shown, using XpYp STELA, that telomere length and heterogeneity does not vary with fetal sex, mode of delivery or sampling site in a healthy pregnancy [24]. In this study, there was no significant relationship (*P* > 0.1) between placental telomere length and variables including maternal BMI, weight gain, socio-economic status, maternal age, gestational age, birth weight and smoking. However, within the control group (n = 69) there was a significant association between placental telomere length and maternal ethnicity (*P* = 0.02), which has been previously described [31].

### Telomere length is associated with *in utero* exposure to poorly controlled hyperglycaemia in male placenta

**Fig 1:**
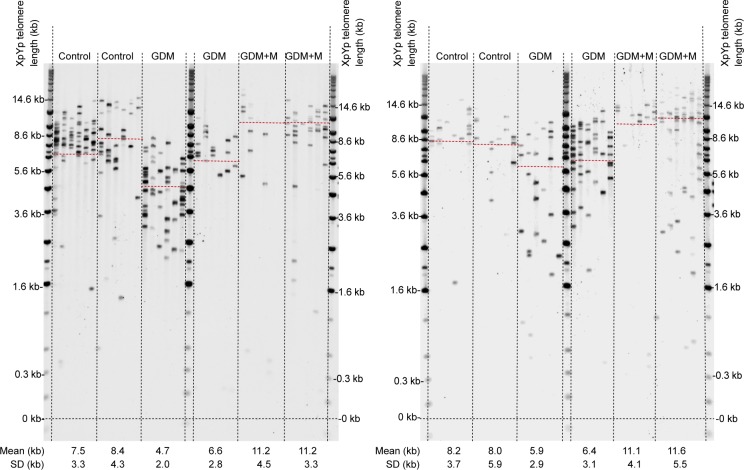
Representative STELA comparing placental samples from control, GDM lifestyle intervention (labelled GDM) and GDM medication (labelled GDM+M) groups. Red dashed line across the STELA profiles indicate the mean. Each sample is analysed with six STELA reactions. Mean telomere lengths are presented below each sample (±SD).

The GDM group included 14 mothers who undertook dietary and exercise changes (lifestyle intervention group) and 17 mothers prescribed insulin and/or metformin (medication group; **Table 2**). Direct observation of the STELA telomere-length profiles identified GDM participants with markedly shortened placental telomeres, which were found to be exclusively from women who did not take medication for GDM (**Fig 1).**

**Table 2:** Maternal and birth characteristics in the lifestyle intervention and the medication group. Mean (SD) is shown. P values were assessed using Student’s t test or Mann–Whitney test.

GDM participants	Lifestyle intervention group (GDM)	Medication group (GDM+M)	*P* VALUE
	N = 14	N = 17	
Maternal age	34 (3.6)	32 (3.9)	0.11
Maternal BMI	30 (8.3)	32 (6.01)	0.38
Weight gain	11.2 (13.0)	10.8 (5.5)	0.93
Birth weight (g)	3734 (503)	3697 (382)	0.81
Gestational age (weeks)	38 (0.60)	38 (0.63)	0.98
Placental weight (g)	691 (149.81)	709 (119.12)	0.70

**Fig 2:**
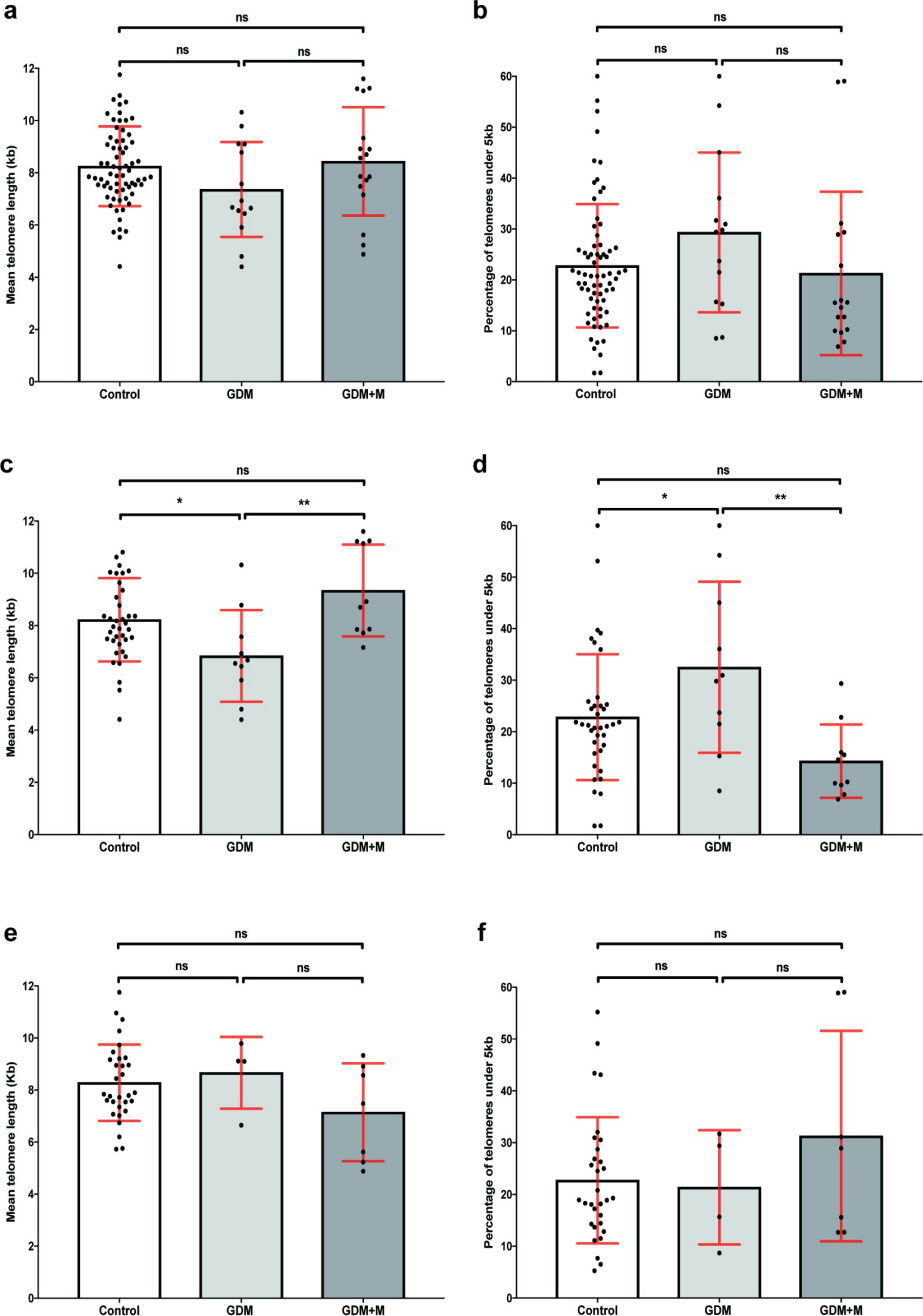
Telomere length differences in placenta from control, GDM and GDM+M groups. Comparison of mean telomere length between control, GDM and GDM+M groups (N = 69+14+17) (a), in male placenta (N = 38+10+10) (c), and in female placenta (N = 31+4+7) (e). Differences in percentage of telomeres under 5kb between control, GDM and GDM+M groups (N = 69+14+17) (b), in male placenta (N = 38+10+10) (d), and in female placenta (N = 31+4+7) (f). Mean telomere length and percentage under 5kb is presented (±SD). ANCOVA test was used to assess statistical significant differences *p<0.05.

Telomeres in the lifestyle intervention group were, on average, 1 kb shorter than those in either the control or medication groups but this was not significant as assessed by ANCOVA (F(2,96) = 1.66, *P = 0*.*2*) **(Fig 2A).** Similarly, there were 7% more telomeres below 5 kb in the lifestyle intervention group but again, without significance **(Fig 2B).** However, when samples where further separated by foetal sex, this revealed significant mean telomere length differences in male placenta between control, GDM lifestyle intervention and GDM medication groups by ANCOVA (F(2,54) = 4.98, *P* = 0.01). Telomeres were shorter in the GDM lifestyle intervention group compared to both the control group (*P* = 0.02) and the GDM medication group (*P* = 0.003) (**Fig 2C**). Consistent with this observation, the percentage of telomeres under 5 kb was about one-third higher in the GDM lifestyle intervention group compared to the control group (*P* = 0.03) and a greater than two-fold compared to the GDM medication group (*P* = 0.004) as assessed by ANCOVA (F(2,54) = 4.65, *P* = 0.01) (**Fig 2D**). For the placenta from female infants, fewer samples were available and no significant differences in telomere length were identified between the three groups (**Fig 2E and 2F**).

## Discussion

There is a growing concern that telomere shortening as a consequence of prenatal adversities such as GDM contributes to the increased occurrence of chronic diseases later in life such as T2DM [32]. In this study, we applied high-resolution telomere length analysis to examine placental telomeres and provide further support for the hypothesis that GDM may exacerbate telomere shortening. We further show that treatment of GDM mothers with metformin and/or insulin is not associated with telomere shortening suggesting that this treatment pathway protects against telomere erosion, an observation with potentially important clinical implications for longer term outcomes.

Telomere length is inherited from the paternal and maternal gametes and consequently at fertilisation each chromosomal telomere starts the new generation at one of two possible lengths. The remarkable telomere heterogeneity we observe in the term placenta arises from an estimated 36 rounds of cell division during gestation [33] indicating a highly dynamic process of telomere maintenance in the placenta. This led us to hypothesise that placental telomeres might provide a sensitive tool for assaying prenatal adversity and, potentially, also measuring the effectiveness of intervention strategies, which we demonstrate here in relation to GDM. We have not shown, in this study, whether the shortened telomeres were inherited by the offspring. While some studies suggest that this is the case [16, 17], other studies find no such association [18–20]. This may be due to the technical challenges imposed by the use of leukocyte DNA. We show here that placental telomere measurements by STELA provides an alternative, sensitive and accurate tool to quantify exposure to GDM.

In this study, we observed shortened telomeres in placenta from women whose GDM was managed only through exercise and diet. We anticipated was that women with more severe GDM, in the medication group, would have the shortest placental telomeres. However, the ten placenta from this group had telomeres in the normal range for a non-diabetic pregnancy. This suggests treatment with metformin/insulin protects against telomere erosion. Metformin has already been proposed as a potential anti-aging drug after experiments in rodents indicated the drug alleviates age-related conditions such as inflammation, oxidative damage and cell senescence [34]. Metformin may function by promoting the upregulation of the telomere repeat-containing ribonucleic acid (TERRA) [35]. Alternatively, metformin may prevent accumulation of senescent (“old”) cells thus decreasing senescent cell abundance as a function of total cell number [36]. A third possibility is that metformin acts indirectly by dampening down the chronic inflammations associated with GDM [37]. Further work is required both to validate our original findings and to explore mechanisms in detail.

Although we have very small numbers of girls from the GDM lifestyle intervention group, data from the four placenta analysed revealed similar profiles to the control and medically treated groups. This is potentially of interest as studies suggest a difference in the response of male and female offspring to diabetic pregnancies. For example, exposure to GDM is a risk factor for childhood overweight in boys, but not in girls [38] [39]. Telomere length in blood leukocytes has been reported to be reduced in girls exposed in utero to GDM but not boys [17]. It may be possible to reconcile these differences if women carrying a male foetus are more likely to take medication, which has been reported [40]. Further research is required.

While the data from our study is exciting, there are limitations. Due to the smaller number of placental samples from girls, we cannot conclude the relationship between telomere length, GDM and medical treatment is restricted to boys. Secondly, while we have statistically controlled for a number of factors which may impact telomere length, questionnaire based studies are inherently subjective and there may be other factors independent of GDM and medical treatment influencing telomere length. As we studied term placenta in individual pregnancies, we do not know when the telomere changes took place and it is possible that the differences in telomere length were inherited.

In summary, we measured telomere length with respect to gestational diabetes diagnosis factoring in foetal sex and treatment pathways. Using STELA we were able to generate high-resolution telomere length profiles from placental samples and we found that placental telomere shortening associated with GDM was prevented by maternal treatment with metformin and/or insulin. It will now be important to expand on these findings to understanding the underlying mechanism and long term consequence of different treatment pathways.

## Supporting information

S1 STROBE checklist (DOCX)

S1 Dataset Data set, related metadata and methods. (PDF)

## Abbreviations

FISH: Fluorescence in situ hybridization
GDM: Gestational diabetes mellitus
STELA: Single telomere length analysis
T2DM: Type 2 diabetes mellitusTweeter

## References

[1] American Diabetes A. Diagnosis and classification of diabetes mellitus. Diabetes Care. 2014;37 Suppl 1:S81–90. Epub 2013/12/21. 10.2337/dc14-S081 . 24357215 10.2337/dc14-S081

[2] Chu SY, Callaghan WM, Kim SY, Schmid CH, Lau J, England LJ, et al Maternal obesity and risk of gestational diabetes mellitus. Diabetes care. 2007;30(8):2070–6. Epub 2007/04/10. 10.2337/dc06-2559a . 17416786 10.2337/dc06-2559a

[3] Brown J, Alwan NA, West J, Brown S, McKinlay CJ, Farrar D, et al Lifestyle interventions for the treatment of women with gestational diabetes. The Cochrane database of systematic reviews. 2017;5:Cd011970 Epub 2017/05/05. 10.1002/14651858.CD011970.pub2 . 28472859 10.1002/14651858.CD011970.pub2 PMC6481373

[4] Hinkle SN, Buck Louis GM, Rawal S, Zhu Y, Albert PS, Zhang C. A longitudinal study of depression and gestational diabetes in pregnancy and the postpartum period. Diabetologia. 2016 10.1007/s00125-016-4086-1 . 27640810 10.1007/s00125-016-4086-1 PMC5101167

[5] Kim C. Maternal outcomes and follow-up after gestational diabetes mellitus. Diabetic medicine: a journal of the British Diabetic Association. 2014;31(3):292–301. Epub 2013/12/18. 10.1111/dme.12382 ; PubMed Central PMCID: PMCPmc3944879. 24341443 10.1111/dme.12382 PMC3944879

[6] Girchenko P, Tuovinen S, Lahti-Pulkkinen M, Lahti J, Savolainen K, Heinonen K, et al Maternal early pregnancy obesity and related pregnancy and pre-pregnancy disorders: associations with child developmental milestones in the prospective PREDO Study. International journal of obesity (2005). 2018. Epub 2018/04/25. 10.1038/s41366-018-0061-x . 29686379 10.1038/s41366-018-0061-x

[7] Fraser A, Lawlor DA. Long-term health outcomes in offspring born to women with diabetes in pregnancy. Current diabetes reports. 2014;14(5):489 Epub 2014/03/26. 10.1007/s11892-014-0489-x ; PubMed Central PMCID: PMCPmc3984422. 24664798 10.1007/s11892-014-0489-x PMC3984422

[8] Holder T, Giannini C, Santoro N, Pierpont B, Shaw M, Duran E, et al A low disposition index in adolescent offspring of mothers with gestational diabetes: a risk marker for the development of impaired glucose tolerance in youth. Diabetologia. 2014;57(11):2413–20. Epub 2014/08/30. 10.1007/s00125-014-3345-2 . 25168408 10.1007/s00125-014-3345-2

[9] Qi Nan W, Ling Z, Bing C. The influence of the telomere-telomerase system on diabetes mellitus and its vascular complications. Expert opinion on therapeutic targets. 2015;19(6):849–64. Epub 2015/02/14. 10.1517/14728222.2015.1016500 . 25677239 10.1517/14728222.2015.1016500

[10] Moyzis RK, Buckingham JM, Cram LS, Dani M, Deaven LL, Jones MD, et al A highly conserved repetitive DNA sequence, (TTAGGG)n, present at the telomeres of human chromosomes. Proc Natl Acad Sci U S A. 1988;85(18):6622–6. Epub 1988/09/01. ; PubMed Central PMCID: PMCPMC282029. 3413114 10.1073/pnas.85.18.6622 PMC282029

[11] Blackburn EH. Telomeres and telomerase: the means to the end (Nobel lecture). Angewandte Chemie. 2010;49(41):7405–21. Epub 2010/09/08. 10.1002/anie.201002387 . 20821774 10.1002/anie.201002387

[12] Blackburn EH. Switching and signaling at the telomere. Cell. 2001;106(6):661–73. Epub 2001/09/27. . 11572773 10.1016/s0092-8674(01)00492-5

[13] Collins K, Mitchell JR. Telomerase in the human organism. Oncogene. 2002;21(4):564–79. Epub 2002/02/19. 10.1038/sj.onc.1205083 . 11850781 10.1038/sj.onc.1205083

[14] Kirchner H, Shaheen F, Kalscheuer H, Schmid SM, Oster H, Lehnert H. The Telomeric Complex and Metabolic Disease. Genes. 2017;8(7). Epub 2017/07/08. 10.3390/genes8070176 ; PubMed Central PMCID: PMCPmc5541309. 28686177 10.3390/genes8070176 PMC5541309

[15] Kuhlow D, Florian S, von Figura G, Weimer S, Schulz N, Petzke KJ, et al Telomerase deficiency impairs glucose metabolism and insulin secretion. Aging. 2010;2(10):650–8. Epub 2010/09/30. doi: 10.18632/aging.100200 ; PubMed Central PMCID: PMCPmc2993795. 20876939 10.18632/aging.100200 PMC2993795

[16] Xu J, Ye J, Wu Y, Zhang H, Luo Q, Han C, et al Reduced fetal telomere length in gestational diabetes. PloS one. 2014;9(1):e86161 Epub 2014/01/28. 10.1371/journal.pone.0086161 ; PubMed Central PMCID: PMCPMC3899117. 24465936 10.1371/journal.pone.0086161 PMC3899117

[17] Hjort L, Vryer R, Grunnet LG, Burgner D, Olsen SF, Saffery R, et al Telomere length is reduced in 9- to 16-year-old girls exposed to gestational diabetes in utero. Diabetologia. 2018;61(4):870–80. Epub 2018/01/25. 10.1007/s00125-018-4549-7 . 29362826 10.1007/s00125-018-4549-7

[18] Harville EW, Williams MA, Qiu CF, Mejia J, Risques RA. Telomere length, pre-eclampsia, and gestational diabetes. BMC Res Notes. 2010;3:113 Epub 2010/04/27. 10.1186/1756-0500-3-113 ; PubMed Central PMCID: PMCPMC2873349. 20416088 10.1186/1756-0500-3-113 PMC2873349

[19] Cross JA, Temple RC, Hughes JC, Dozio NC, Brennan C, Stanley K, et al Cord blood telomere length, telomerase activity and inflammatory markers in pregnancies in women with diabetes or gestational diabetes. Diabet Med. 2010;27(11):1264–70. Epub 2010/10/19. 10.1111/j.1464-5491.2010.03099.x . 20950384 10.1111/j.1464-5491.2010.03099.x

[20] Biron-Shental T, Liberman M, Elbaz M, Laish I, Sharony R, Amiel A. Telomere homeostasis in placentas from pregnancies with uncontrolled diabetes. Placenta. 2016;44:13–8. Epub 2016/07/28. 10.1016/j.placenta.2016.05.009 . 27452433 10.1016/j.placenta.2016.05.009

[21] Adalsteinsson BT, Gudnason H, Aspelund T, Harris TB, Launer LJ, Eiriksdottir G, et al Heterogeneity in white blood cells has potential to confound DNA methylation measurements. PloS one. 2012;7(10):e46705 Epub 2012/10/17. 10.1371/journal.pone.0046705 ; PubMed Central PMCID: PMCPMC3465258. 23071618 10.1371/journal.pone.0046705 PMC3465258

[22] John R, Hemberger M. A placenta for life. Reprod Biomed Online. 2012;25(1):5–11. Epub 2012/05/15. S1472-6483(12)00206-4 [pii] 10.1016/j.rbmo.2012.03.018 . 22578825 10.1016/j.rbmo.2012.03.018

[23] Biron-Shental T, Sukenik-Halevy R, Naboani H, Liberman M, Kats R, Amiel A. Telomeres are shorter in placentas from pregnancies with uncontrolled diabetes. Placenta. 2015;36(2):199–203. Epub 2014/12/17. 10.1016/j.placenta.2014.11.011 . 25499309 10.1016/j.placenta.2014.11.011

[24] Garcia-Martin I, Janssen AB, Jones RE, Grimstead JW, Penketh RJA, Baird DM, et al Telomere length heterogeneity in placenta revealed with high-resolution telomere length analysis. Placenta. 2017;59:61–8. Epub 2017/11/08. 10.1016/j.placenta.2017.09.007 ; PubMed Central PMCID: PMCPMC5687939. 29108638 10.1016/j.placenta.2017.09.007 PMC5687939

[25] Baird DM, Rowson J, Wynford-Thomas D, Kipling D. Extensive allelic variation and ultrashort telomeres in senescent human cells. Nat Genet. 2003;33(2):203–7. 10.1038/ng1084 . 12539050 10.1038/ng1084

[26] Janssen AB, Tunster SJ, Savory N, Holmes A, Beasley J, Parveen SA, et al Placental expression of imprinted genes varies with sampling site and mode of delivery. Placenta. 2015;36(8):790–5. 10.1016/j.placenta.2015.06.011 ; PubMed Central PMCID: PMCPMC4535278. 26162698 10.1016/j.placenta.2015.06.011 PMC4535278

[27] Capper R, Britt-Compton B, Tankimanova M, Rowson J, Letsolo B, Man S, et al The nature of telomere fusion and a definition of the critical telomere length in human cells. Genes & development. 2007;21(19):2495–508. Epub 2007/10/03. 10.1101/gad.439107 ; PubMed Central PMCID: PMCPMC1993879. 17908935 10.1101/gad.439107 PMC1993879

[28] Yuen L, Wong VW. Gestational diabetes mellitus: Challenges for different ethnic groups. World J Diabetes. 2015;6(8):1024–32. Epub 2015/08/05. 10.4239/wjd.v6.i8.1024 ; PubMed Central PMCID: PMCPMC4515442. 26240699 10.4239/wjd.v6.i8.1024 PMC4515442

[29] Lao TT, Ho LF, Chan BC, Leung WC. Maternal age and prevalence of gestational diabetes mellitus. Diabetes care. 2006;29(4):948–9. Epub 2006/03/29. . 16567851 10.2337/diacare.29.04.06.dc05-2568

[30] Sebire NJ, Jolly M, Harris JP, Wadsworth J, Joffe M, Beard RW, et al Maternal obesity and pregnancy outcome: a study of 287,213 pregnancies in London. Int J Obes Relat Metab Disord. 2001;25(8):1175–82. Epub 2001/07/31. 10.1038/sj.ijo.0801670 . 11477502 10.1038/sj.ijo.0801670

[31] Jones CW, Gambala C, Esteves KC, Wallace M, Schlesinger R, O'Quinn M, et al Differences in placental telomere length suggest a link between racial disparities in birth outcomes and cellular aging. Am J Obstet Gynecol. 2017;216(3):294 e1– e8. Epub 2016/11/21. 10.1016/j.ajog.2016.11.1027 ; PubMed Central PMCID: PMCPMC5334179. 27865975 10.1016/j.ajog.2016.11.1027 PMC5334179

[32] Entringer S, de Punder K, Buss C, Wadhwa PD. The fetal programming of telomere biology hypothesis: an update. Philosophical transactions of the Royal Society of London Series B, Biological sciences. 2018;373(1741). Epub 2018/01/18. 10.1098/rstb.2017.0151 ; PubMed Central PMCID: PMCPMC5784074. 29335381 10.1098/rstb.2017.0151 PMC5784074

[33] Simpson RA, Mayhew TM, Barnes PR. From 13 weeks to term, the trophoblast of human placenta grows by the continuous recruitment of new proliferative units: a study of nuclear number using the disector. Placenta. 1992;13(5):501–12. Epub 1992/09/01. . 1470609 10.1016/0143-4004(92)90055-x

[34] Menendez JA, Cufi S, Oliveras-Ferraros C, Vellon L, Joven J, Vazquez-Martin A. Gerosuppressant metformin: less is more. Aging. 2011;3(4):348–62. Epub 2011/04/13. doi: 10.18632/aging.100316 ; PubMed Central PMCID: PMCPMC3117449. 21483040 10.18632/aging.100316 PMC3117449

[35] Diman A, Boros J, Poulain F, Rodriguez J, Purnelle M, Episkopou H, et al Nuclear respiratory factor 1 and endurance exercise promote human telomere transcription. Sci Adv. 2016;2(7):e1600031 Epub 2016/11/08. 10.1126/sciadv.1600031 ; PubMed Central PMCID: PMCPMC5087959. 27819056 10.1126/sciadv.1600031 PMC5087959

[36] Anisimov VN, Berstein LM, Popovich IG, Zabezhinski MA, Egormin PA, Piskunova TS, et al If started early in life, metformin treatment increases life span and postpones tumors in female SHR mice. Aging. 2011;3(2):148–57. Epub 2011/03/10. doi: 10.18632/aging.100273 ; PubMed Central PMCID: PMCPMC3082009. 21386129 10.18632/aging.100273 PMC3082009

[37] Saisho Y. Metformin and Inflammation: Its Potential Beyond Glucose-lowering Effect. Endocr Metab Immune Disord Drug Targets. 2015;15(3):196–205. Epub 2015/03/17. . 25772174 10.2174/1871530315666150316124019

[38] Le Moullec N, Fianu A, Maillard O, Chazelle E, Naty N, Schneebeli C, et al Sexual dimorphism in the association between gestational diabetes mellitus and overweight in offspring at 5–7 years: The OBEGEST cohort study. PloS one. 2018;13(4):e0195531 Epub 2018/04/06. 10.1371/journal.pone.0195531 ; PubMed Central PMCID: PMCPMC5886576. 29621322 10.1371/journal.pone.0195531 PMC5886576

[39] Li S, Zhu Y, Yeung E, Chavarro JE, Yuan C, Field AE, et al Offspring risk of obesity in childhood, adolescence and adulthood in relation to gestational diabetes mellitus: a sex-specific association. Int J Epidemiol. 2017;46(5):1533–41. Epub 2017/10/13. 10.1093/ije/dyx151 ; PubMed Central PMCID: PMCPMC5837775. 29024955 10.1093/ije/dyx151 PMC5837775

[40] Giannubilo SR, Pasculli A, Ballatori C, Biagini A, Ciavattini A. Fetal Sex, Need for Insulin, and Perinatal Outcomes in Gestational Diabetes Mellitus: An Observational Cohort Study. Clin Ther. 2018;40(4):587–92. Epub 2018/03/24. 10.1016/j.clinthera.2018.02.015 . 29567300 10.1016/j.clinthera.2018.02.015

