# Supplementary material for: Metformin and insulin treatment prevent placental telomere attrition in boys exposed to maternal diabetes
Source: PLoS One. 2018 Dec 11;13(12):e0208533. doi: 10.1371/journal.pone.0208533 (PMC6289439; doi:10.1371/journal.pone.0208533)
Supplement: S1 Dataset — (PDF) [file pone.0208533.s002.pdf]

```
SORT CASES  BY Groups_statistics
SPLIT FILE LAYERED BY Groups_statistics
FREQUENCIES VARIABLES=Mean_TL
  /ORDER=ANALYSIS.
```

Frequencies

Notes

Output Created		27-NOV-2018 12:20:25
Comments		
Input	Data	C:\Users\c1571923\Desktop\GDM project\100 gdm a paper+median.sav
	Active Dataset	DataSet1
	Filter	<none>
	Weight	<none>
	Split File	Groups_statistics
	N of Rows in Working Data File	100
Missing Value Handling	Definition of Missing	User-defined missing values are treated as missing.
	Cases Used	Statistics are based on all cases with valid data.
Syntax		FREQUENCIES VARIABLES=Mean_TL /ORDER=ANALYSIS.
Resources	Processor Time	00:00:00.02
	Elapsed Time	00:00:00.02

Statistics

Mean telomere length

control	N	Valid	69
		Missing	0
gdm	N	Valid	14
		Missing	0
gdm+m	N	Valid	17
		Missing	0

Mean telomere length

Groups_statistics			Frequency	Percent	Valid Percent	Cumulative Percent
control	Valid	4.41	1	1.4	1.4	1.4
		5.53	1	1.4	1.4	2.9
		5.73	1	1.4	1.4	4.3
		5.76	1	1.4	1.4	5.8
		5.83	1	1.4	1.4	7.2
		6.20	1	1.4	1.4	8.7
		6.55	1	1.4	1.4	10.1
		6.58	1	1.4	1.4	11.6
		6.74	1	1.4	1.4	13.0
		6.81	1	1.4	1.4	14.5
		6.95	1	1.4	1.4	15.9
		7.00	1	1.4	1.4	17.4
		7.02	1	1.4	1.4	18.8
		7.07	1	1.4	1.4	20.3
		7.19	1	1.4	1.4	21.7
		7.29	1	1.4	1.4	23.2
		7.35	1	1.4	1.4	24.6
		7.42	1	1.4	1.4	26.1
		7.46	1	1.4	1.4	27.5
		7.49	1	1.4	1.4	29.0
		7.54	1	1.4	1.4	30.4
		7.54	1	1.4	1.4	31.9
		7.55	1	1.4	1.4	33.3
		7.58	2	2.9	2.9	36.2
		7.61	1	1.4	1.4	37.7
		7.62	1	1.4	1.4	39.1
		7.72	1	1.4	1.4	40.6
		7.75	1	1.4	1.4	42.0
		7.76	1	1.4	1.4	43.5
		7.79	1	1.4	1.4	44.9
		7.84	1	1.4	1.4	46.4
		7.85	1	1.4	1.4	47.8
		7.88	1	1.4	1.4	49.3
		7.90	1	1.4	1.4	50.7
		7.96	1	1.4	1.4	52.2
		8.09	1	1.4	1.4	53.6
		8.18	1	1.4	1.4	55.1
		8.24	1	1.4	1.4	56.5
		8.26	1	1.4	1.4	58.0
		8.36	1	1.4	1.4	59.4
		8.36	1	1.4	1.4	60.9
		8.36	1	1.4	1.4	62.3

Mean telomere length

Groups_statistics			Frequency	Percent	Valid Percent	Cumulative Percent
		8.44	1	1.4	1.4	63.8
		8.60	1	1.4	1.4	65.2
		8.77	1	1.4	1.4	66.7
		8.93	1	1.4	1.4	68.1
		8.95	1	1.4	1.4	69.6
		8.96	1	1.4	1.4	71.0
		9.08	1	1.4	1.4	72.5
		9.17	1	1.4	1.4	73.9
		9.21	1	1.4	1.4	75.4
		9.24	1	1.4	1.4	76.8
		9.35	1	1.4	1.4	78.3
		9.46	1	1.4	1.4	79.7
		9.64	1	1.4	1.4	81.2
		9.73	1	1.4	1.4	82.6
		10.00	1	1.4	1.4	84.1
		10.01	1	1.4	1.4	85.5
		10.04	1	1.4	1.4	87.0
		10.09	1	1.4	1.4	88.4
		10.27	1	1.4	1.4	89.9
		10.30	1	1.4	1.4	91.3
		10.62	1	1.4	1.4	92.8
		10.71	1	1.4	1.4	94.2
		10.81	1	1.4	1.4	95.7
		10.96	1	1.4	1.4	97.1
		11.76	1	1.4	1.4	98.6
		12.37	1	1.4	1.4	100.0
		Total	69	100.0	100.0	
gdm	Valid	4.40	1	7.1	7.1	7.1
		4.80	1	7.1	7.1	14.3
		5.91	1	7.1	7.1	21.4
		6.44	1	7.1	7.1	28.6
		6.55	1	7.1	7.1	35.7
		6.65	1	7.1	7.1	42.9
		6.68	1	7.1	7.1	50.0
		6.93	1	7.1	7.1	57.1
		7.57	1	7.1	7.1	64.3
		8.78	1	7.1	7.1	71.4
		9.10	1	7.1	7.1	78.6
		9.11	1	7.1	7.1	85.7
		9.79	1	7.1	7.1	92.9
		10.32	1	7.1	7.1	100.0
		Total	14	100.0	100.0	

Mean telomere length

Groups_statistics			Frequency	Percent	Valid Percent	Cumulative Percent
gdm+m	Valid	4.88	1	5.9	5.9	5.9
		5.23	1	5.9	5.9	11.8
		5.62	1	5.9	5.9	17.6
		7.16	1	5.9	5.9	23.5
		7.48	1	5.9	5.9	29.4
		7.72	1	5.9	5.9	35.3
		7.85	1	5.9	5.9	41.2
		7.86	1	5.9	5.9	47.1
		8.57	1	5.9	5.9	52.9
		8.70	1	5.9	5.9	58.8
		8.91	1	5.9	5.9	64.7
		8.92	1	5.9	5.9	70.6
		9.33	1	5.9	5.9	76.5
		11.14	1	5.9	5.9	82.4
		11.22	1	5.9	5.9	88.2
		11.24	1	5.9	5.9	94.1
		11.60	1	5.9	5.9	100.0
		Total	17	100.0	100.0	

```
FREQUENCIES VARIABLES=Telomere_percentage
  /ORDER=ANALYSIS.
```

Frequencies

Notes

Output Created		27-NOV-2018 12:20:48
Comments		
Input	Data	C:\Users\c1571923\Desktop\GDM project\100 gdm a paper+median.sav
	Active Dataset	DataSet1
	Filter	<none>
	Weight	<none>
	Split File	Groups_statistics
	N of Rows in Working Data File	100
Missing Value Handling	Definition of Missing	User-defined missing values are treated as missing.
	Cases Used	Statistics are based on all cases with valid data.
Syntax		FREQUENCIES VARIABLES=Telomere_percentage /ORDER=ANALYSIS.
Resources	Processor Time	00:00:00.00
	Elapsed Time	00:00:00.00

Statistics

Lower 5kb

control	N	Valid	69
		Missing	0
gdm	N	Valid	14
		Missing	0
gdm+m	N	Valid	17
		Missing	0

Lower 5kb

Groups_statistics			Frequency	Percent	Valid Percent	Cumulative Percent
control	Valid	1.72	1	1.4	1.4	1.4
		1.75	1	1.4	1.4	2.9
		5.26	1	1.4	1.4	4.3
		6.52	1	1.4	1.4	5.8
		7.69	1	1.4	1.4	7.2
		7.93	1	1.4	1.4	8.7
		8.30	1	1.4	1.4	10.1
		10.71	1	1.4	1.4	11.6
		10.81	1	1.4	1.4	13.0
		11.11	1	1.4	1.4	14.5
		11.53	1	1.4	1.4	15.9
		12.35	1	1.4	1.4	17.4
		12.85	1	1.4	1.4	18.8
		13.33	1	1.4	1.4	20.3
		13.69	1	1.4	1.4	21.7
		14.28	1	1.4	1.4	23.2
		14.45	1	1.4	1.4	24.6
		15.78	1	1.4	1.4	26.1
		16.00	1	1.4	1.4	27.5
		16.32	1	1.4	1.4	29.0
		17.24	1	1.4	1.4	30.4
		17.39	1	1.4	1.4	31.9
		17.94	1	1.4	1.4	33.3
		18.07	1	1.4	1.4	34.8
		18.18	1	1.4	1.4	36.2
		18.30	1	1.4	1.4	37.7
		18.88	1	1.4	1.4	39.1
		18.94	1	1.4	1.4	40.6
		19.29	2	2.9	2.9	43.5
		19.31	1	1.4	1.4	44.9
		20.23	1	1.4	1.4	46.4
		20.75	2	2.9	2.9	49.3
		20.79	1	1.4	1.4	50.7
		21.05	1	1.4	1.4	52.2
		21.25	1	1.4	1.4	53.6
		21.42	2	2.9	2.9	56.5
		21.87	2	2.9	2.9	59.4
		23.40	1	1.4	1.4	60.9
		24.41	1	1.4	1.4	62.3
		24.44	1	1.4	1.4	63.8
		24.52	1	1.4	1.4	65.2
		25.00	3	4.3	4.3	69.6

Lower 5kb

Groups_statistics			Frequency	Percent	Valid Percent	Cumulative Percent
		25.30	1	1.4	1.4	71.0
		25.67	1	1.4	1.4	72.5
		25.86	1	1.4	1.4	73.9
		26.31	1	1.4	1.4	75.4
		26.66	1	1.4	1.4	76.8
		26.86	1	1.4	1.4	78.3
		28.71	1	1.4	1.4	79.7
		30.55	1	1.4	1.4	81.2
		30.95	1	1.4	1.4	82.6
		32.05	1	1.4	1.4	84.1
		35.95	1	1.4	1.4	85.5
		37.34	1	1.4	1.4	87.0
		38.09	1	1.4	1.4	88.4
		39.13	1	1.4	1.4	89.9
		39.72	1	1.4	1.4	91.3
		43.13	1	1.4	1.4	92.8
		43.39	1	1.4	1.4	94.2
		49.15	1	1.4	1.4	95.7
		53.12	1	1.4	1.4	97.1
		55.22	1	1.4	1.4	98.6
		60.00	1	1.4	1.4	100.0
		Total	69	100.0	100.0	
gdm	Valid	8.51	1	7.1	7.1	7.1
		8.69	1	7.1	7.1	14.3
		15.27	1	7.1	7.1	21.4
		15.68	1	7.1	7.1	28.6
		21.50	1	7.1	7.1	35.7
		23.68	1	7.1	7.1	42.9
		29.41	1	7.1	7.1	50.0
		29.82	1	7.1	7.1	57.1
		30.95	1	7.1	7.1	64.3
		31.70	1	7.1	7.1	71.4
		36.05	1	7.1	7.1	78.6
		45.05	1	7.1	7.1	85.7
		54.25	1	7.1	7.1	92.9
		60.00	1	7.1	7.1	100.0
		Total	14	100.0	100.0	
gdm+m	Valid	6.89	1	5.9	5.9	5.9
		7.81	1	5.9	5.9	11.8
		9.67	1	5.9	5.9	17.6
		10.00	1	5.9	5.9	23.5
		10.25	1	5.9	5.9	29.4

Lower 5kb

Groups_statistics	Frequency	Percent	Valid Percent	Cumulative Percent
12.69	1	5.9	5.9	35.3
12.72	1	5.9	5.9	41.2
14.58	1	5.9	5.9	47.1
15.51	1	5.9	5.9	52.9
15.58	1	5.9	5.9	58.8
16.00	1	5.9	5.9	64.7
22.80	1	5.9	5.9	70.6
28.91	1	5.9	5.9	76.5
29.34	1	5.9	5.9	82.4
31.11	1	5.9	5.9	88.2
58.90	1	5.9	5.9	94.1
59.06	1	5.9	5.9	100.0
Total	17	100.0	100.0	

```
 SPLIT FILE OFF.
UNIANOVA Mean_TL BY Groups_statistics WITH Caucasian_yes_no
  /METHOD=SSTYPE(3)
  /INTERCEPT=INCLUDE
  /EMMEANS=TABLES(Groups_statistics) WITH(Caucasian_yes_no=MEAN) COMPARE AD
J(LSD)
  /PRINT=DESCRIPTIVE
  /CRITERIA=ALPHA(.05)
  /DESIGN=Caucasian_yes_no Groups_statistics.
```

Univariate Analysis of Variance

Notes

Output Created		27-NOV-2018 12:21:15
Comments		
Input	Data	C:\Users\c1571923\Desktop\GDM project\100 gdm a paper+median.sav
	Active Dataset	DataSet1
	Filter	<none>
	Weight	<none>
	Split File	<none>
	N of Rows in Working Data File	100
Missing Value Handling	Definition of Missing	User-defined missing values are treated as missing.
	Cases Used	Statistics are based on all cases with valid data for all variables in the model.
Syntax		UNIANOVA Mean_TL BY Groups_statistics WITH Caucasian_yes_no<br>/METHOD=SSTYPE(3)<br>/INTERCEPT=INCLUDE<br>/EMMEANS=TABLES (Groups_statistics) WITH (Caucasian_yes_no=MEAN) COMPARE ADJ(LSD)<br>/PRINT=DESCRIPTIVE<br>/CRITERIA=ALPHA(.05)<br>/DESIGN=Caucasian_yes_no Groups_statistics.
Resources	Processor Time	00:00:00.00
	Elapsed Time	00:00:00.00

Between-Subjects Factors

		Value Label	N
Groups_statistics	1.00	control	69
	2.00	gdm	14
	3.00	gdm+m	17

Descriptive Statistics

Dependent Variable: Mean telomere length

Groups_statistics	Mean	Std. Deviation	N
control	8.2480	1.52686	69
gdm	7.3589	1.81684	14
gdm+m	8.4367	2.07377	17
Total	8.1556	1.68495	100

Tests of Between-Subjects Effects

Dependent Variable: Mean telomere length

Source	Type III Sum of Squares	df	Mean Square	F	Sig.
Corrected Model	23.961[a]	3	7.987	2.982	.035
Intercept	319.704	1	319.704	119.374	.000
Caucasian_yes_no	13.143	1	13.143	4.907	.029
Groups_statistics	8.935	2	4.468	1.668	.194
Error	257.105	96	2.678		
Total	6932.474	100			
Corrected Total	281.067	99			

a. R Squared = .085 (Adjusted R Squared = .057)

Estimated Marginal Means

Groups_statistics

Estimates

Dependent Variable: Mean telomere length

Groups_statistics	Mean	Std. Error	95% Confidence Interval	
			Lower Bound	Upper Bound
control	8.269[a]	.197	7.877	8.661
gdm	7.414[a]	.438	6.544	8.283
gdm+m	8.306[a]	.401	7.510	9.103

a. Covariates appearing in the model are evaluated at the following values: Caucasian_yes_no = 1.1200.

Pairwise Comparisons

Dependent Variable: Mean telomere length

(I) Groups_statistics	(J) Groups_statistics	Mean Difference (I-J)	Std. Error	Sig.[a]	95% Confidence ...[a]
					Lower Bound
control	gdm	.855	.480	.078	-.098
	gdm+m	-.037	.448	.934	-.927
gdm	control	-.855	.480	.078	-1.808
	gdm+m	-.892	.597	.138	-2.077
gdm+m	control	.037	.448	.934	-.853
	gdm	.892	.597	.138	-.292

Pairwise Comparisons

Dependent Variable: Mean telomere length

(I) Groups_statistics	(J) Groups_statistics	95% Confidence Interval for ...[a] Upper Bound
control	gdm	1.808
	gdm+m	.853
gdm	control	.098
	gdm+m	.292
gdm+m	control	.927
	gdm	2.077

Based on estimated marginal means

a. Adjustment for multiple comparisons: Least Significant Difference (equivalent to no adjustments).

Univariate Tests

Dependent Variable: Mean telomere length

	Sum of Squares	df	Mean Square	F	Sig.
Contrast	8.935	2	4.468	1.668	.194
Error	257.105	96	2.678		

The F tests the effect of Groups_statistics. This test is based on the linearly independent pairwise comparisons among the estimated marginal means.

```
UNIANOVA Telomere_percentage BY Groups_statistics WITH Caucasian_yes_no
  /METHOD=SSTYPE(3)
  /INTERCEPT=INCLUDE
  /EMMEANS=TABLES(Groups_statistics) WITH(Caucasian_yes_no=MEAN) COMPARE AD
J(LSD)
  /PRINT=DESCRIPTIVE
  /CRITERIA=ALPHA(.05)
  /DESIGN=Caucasian_yes_no Groups_statistics.
```

Univariate Analysis of Variance

Notes

Output Created		27-NOV-2018 12:21:31
Comments		
Input	Data	C:\Users\c1571923\Desktop\GDM project\100 gdm a paper+median.sav
	Active Dataset	DataSet1
	Filter	<none>
	Weight	<none>
	Split File	<none>
	N of Rows in Working Data File	100
Missing Value Handling	Definition of Missing	User-defined missing values are treated as missing.
	Cases Used	Statistics are based on all cases with valid data for all variables in the model.
Syntax		UNIANOVA Telomere_percentage BY Groups_statistics WITH Caucasian_yes_no<br>/METHOD=SSTYPE(3)<br>/INTERCEPT=INCLUDE<br>/EMMEANS=TABLES (Groups_statistics) WITH (Caucasian_yes_no=MEAN) COMPARE ADJ(LSD)<br>/PRINT=DESCRIPTIVE<br>/CRITERIA=ALPHA(.05)<br>/DESIGN=Caucasian_yes_no Groups_statistics.
Resources	Processor Time	00:00:00.02
	Elapsed Time	00:00:00.02

Between-Subjects Factors

		Value Label	N
Groups_statistics	1.00	control	69
	2.00	gdm	14
	3.00	gdm+m	17

Descriptive Statistics

Dependent Variable: Lower 5kb

Groups_statistics	Mean	Std. Deviation	N
control	22.7759	12.11071	69
gdm	29.3257	15.68718	14
gdm+m	21.2835	16.05173	17
Total	23.4392	13.44260	100

Tests of Between-Subjects Effects

Dependent Variable: Lower 5kb

Source	Type III Sum of Squares	df	Mean Square	F	Sig.
Corrected Model	733.065[a]	3	244.355	1.367	.257
Intercept	5787.574	1	5787.574	32.384	.000
Caucasian_yes_no	138.599	1	138.599	.776	.381
Groups_statistics	540.276	2	270.138	1.512	.226
Error	17156.577	96	178.714		
Total	72829.251	100			
Corrected Total	17889.642	99			

a. R Squared = .041 (Adjusted R Squared = .011)

Estimated Marginal Means

Groups_statistics

Estimates

Dependent Variable: Lower 5kb

Groups_statistics	Mean	Std. Error	95% Confidence Interval	
			Lower Bound	Upper Bound
control	22.708[a]	1.611	19.510	25.906
gdm	29.147[a]	3.579	22.044	36.251
gdm+m	21.707[a]	3.278	15.201	28.213

a. Covariates appearing in the model are evaluated at the following values: Caucasian_yes_no = 1.1200.

Pairwise Comparisons

Dependent Variable: Lower 5kb

(I) Groups_statistics	(J) Groups_statistics	Mean Difference (I-J)	Std. Error	Sig.[a]	95% Confidence ...[a]
					Lower Bound
control	gdm	-6.440	3.921	.104	-14.222
	gdm+m	1.001	3.663	.785	-6.269
gdm	control	6.440	3.921	.104	-1.343
	gdm+m	7.440	4.873	.130	-2.232
gdm+m	control	-1.001	3.663	.785	-8.271
	gdm	-7.440	4.873	.130	-17.113

Pairwise Comparisons

Dependent Variable: Lower 5kb

(I) Groups_statistics	(J) Groups_statistics	95% Confidence Interval for ...[a] Upper Bound
control	gdm	1.343
	gdm+m	8.271
gdm	control	14.222
	gdm+m	17.113
gdm+m	control	6.269
	gdm	2.232

Based on estimated marginal means

a. Adjustment for multiple comparisons: Least Significant Difference (equivalent to no adjustments).

Univariate Tests

Dependent Variable: Lower 5kb

	Sum of Squares	df	Mean Square	F	Sig.
Contrast	540.276	2	270.138	1.512	.226
Error	17156.577	96	178.714		

The F tests the effect of Groups_statistics. This test is based on the linearly independent pairwise comparisons among the estimated marginal means.

```
SORT CASES BY Fetal_sex.
SPLIT FILE LAYERED BY Fetal_sex.
UNIANOVA Mean_TL BY Groups_statistics WITH Caucasian_yes_no
  /METHOD=SSTYPE(3)
  /INTERCEPT=INCLUDE
  /EMMEANS=TABLES(Groups_statistics) WITH(Caucasian_yes_no=MEAN) COMPARE AD
J(LSD)
  /PRINT=DESCRIPTIVE
  /CRITERIA=ALPHA(.05)
  /DESIGN=Caucasian_yes_no Groups_statistics.
```

Univariate Analysis of Variance

Notes

Output Created		27-NOV-2018 12:22:10
Comments		
Input	Data	C:\Users\c1571923\Desktop\GDM project\100 gdm a paper+median. sav
	Active Dataset	DataSet1
	Filter	<none>
	Weight	<none>
	Split File	Recorded in notes: Fetal Sex
	N of Rows in Working Data File	100
Missing Value Handling	Definition of Missing	User-defined missing values are treated as missing.
	Cases Used	Statistics are based on all cases with valid data for all variables in the model.
Syntax		UNIANOVA Mean_TL BY Groups_statistics WITH Caucasian_yes_no<br>/METHOD=SSTYPE(3)<br>/INTERCEPT=INCLUDE<br>/EMMEANS=TABLES (Groups_statistics) WITH (Caucasian_yes_no=MEAN) COMPARE ADJ(LSD)<br>/PRINT=DESCRIPTIVE<br>/CRITERIA=ALPHA(.05)<br>/DESIGN=Caucasian_yes_no Groups_statistics.
Resources	Processor Time	00:00:00.03
	Elapsed Time	00:00:00.03

Between-Subjects Factors

Recorded in notes: Fetal Sex			Value Label	N
Male	Groups_statistics	1.00	control	38
		2.00	gdm	10
		3.00	gdm+m	10
Female	Groups_statistics	1.00	control	31
		2.00	gdm	4
		3.00	gdm+m	7

Descriptive Statistics

Dependent Variable: Mean telomere length

Recorded in notes: Fetal Sex	Groups_statistics	Mean	Std. Deviation	N
Male	control	8.2209	1.59329	38
	gdm	6.8378	1.75435	10
	gdm+m	9.3402	1.75834	10
	Total	8.1754	1.78193	58
Female	control	8.2812	1.46673	31
	gdm	8.6618	1.37923	4
	gdm+m	7.1460	1.87787	7
	Total	8.1283	1.56191	42

Tests of Between-Subjects Effects

Dependent Variable: Mean telomere length

Recorded in notes: Fetal Sex	Source	Type III Sum of Squares	df	Mean Square
Male	Corrected Model	40.338[a]	3	13.446
	Intercept	197.673	1	197.673
	Caucasian_yes_no	8.800	1	8.800
	Groups_statistics	25.990	2	12.995
	Error	140.652	54	2.605
	Total	4057.557	58	
	Corrected Total	180.990	57	
Female	Corrected Model	11.353[b]	3	3.784
	Intercept	134.120	1	134.120
	Caucasian_yes_no	2.735	1	2.735
	Groups_statistics	9.485	2	4.742
	Error	88.669	38	2.333
	Total	2874.916	42	
	Corrected Total	100.022	41	

Tests of Between-Subjects Effects

Dependent Variable: Mean telomere length

Recorded in notes: Fetal Sex	Source	F	Sig.
Male	Corrected Model	5.162	.003
	Intercept	75.892	.000
	Caucasian_yes_no	3.379	.072
	Groups_statistics	4.989	.010
	Error		
	Total		
	Corrected Total		
Female	Corrected Model	1.622	.200
	Intercept	57.478	.000
	Caucasian_yes_no	1.172	.286
	Groups_statistics	2.032	.145
	Error		
	Total		
	Corrected Total		

a. R Squared = .223 (Adjusted R Squared = .180)

b. R Squared = .114 (Adjusted R Squared = .044)

Estimated Marginal Means

Groups_statistics

Estimates

Dependent Variable: Mean telomere length

Recorded in notes: Fetal Sex	Groups_statistics	Mean	Std. Error	95% Confidence Interval	
				Lower Bound	Upper Bound
Male	control	8.259[a]	.263	7.732	8.785
	gdm	6.882[a]	.511	5.857	7.906
	gdm+m	9.153[a]	.520	8.109	10.196
Female	control	8.280[b]	.274	7.724	8.835
	gdm	8.745[b]	.768	7.191	10.299
	gdm+m	7.104[b]	.579	5.933	8.276

a. Covariates appearing in the model are evaluated at the following values: Caucasian_yes_no = 1.1379.

b. Covariates appearing in the model are evaluated at the following values: Caucasian_yes_no = 1.0952.

Pairwise Comparisons

Dependent Variable: Mean telomere length

Recorded in notes: Fetal Sex	(I) Groups_statistics	(J) Groups_statistics	Mean Difference (I-J)	Std. Error
Male	control	gdm	1.377*	.574
		gdm+m	-.894	.587
	gdm	control	-1.377*	.574
		gdm+m	-2.271*	.733
	gdm+m	control	.894	.587
		gdm	2.271*	.733
Female	control	gdm	-.465	.815
		gdm+m	1.176	.640
	gdm	control	.465	.815
		gdm+m	1.641	.964
	gdm+m	control	-1.176	.640
		gdm	-1.641	.964

Pairwise Comparisons

Dependent Variable: Mean telomere length

Recorded in notes: Fetal Sex	(I) Groups_statistics	(J) Groups_statistics	Sig.[b]	95% Confidence ...[b]
				Lower Bound
Male	control	gdm	.020	.227
		gdm+m	.133	-2.070
	gdm	control	.020	-2.527
		gdm+m	.003	-3.740
	gdm+m	control	.133	-.282
		gdm	.003	.802
Female	control	gdm	.572	-2.116
		gdm+m	.074	-.121
	gdm	control	.572	-1.185
		gdm+m	.097	-.311
	gdm+m	control	.074	-2.472
		gdm	.097	-3.593

Pairwise Comparisons

Dependent Variable: Mean telomere length

Recorded in notes: Fetal Sex	(I) Groups_statistics	(J) Groups_statistics	95% Confidence Interval for ...[b] Upper Bound
Male	control	gdm	2.527
		gdm+m	.282
	gdm	control	-.227
		gdm+m	-.802
	gdm+m	control	2.070
		gdm	3.740
Female	control	gdm	1.185
		gdm+m	2.472
	gdm	control	2.116
		gdm+m	3.593
	gdm+m	control	.121
		gdm	.311

Based on estimated marginal means

*. The mean difference is significant at the .05 level.

b. Adjustment for multiple comparisons: Least Significant Difference (equivalent to no adjustments).

Univariate Tests

Dependent Variable: Mean telomere length

Recorded in notes: Fetal Sex		Sum of Squares	df	Mean Square	F	Sig.
Male	Contrast	25.990	2	12.995	4.989	.010
	Error	140.652	54	2.605		
Female	Contrast	9.485	2	4.742	2.032	.145
	Error	88.669	38	2.333		

The F tests the effect of Groups_statistics. This test is based on the linearly independent pairwise comparisons among the estimated marginal means.

```
UNIANOVA Telomere_percentage BY Groups_statistics WITH Caucasian_yes_no
  /METHOD=SSTYPE(3)
  /INTERCEPT=INCLUDE
  /EMMEANS=TABLES(Groups_statistics) WITH(Caucasian_yes_no=MEAN) COMPARE AD
J(LSD)
  /PRINT=DESCRIPTIVE
  /CRITERIA=ALPHA(.05)
  /DESIGN=Caucasian_yes_no Groups_statistics.
```

Univariate Analysis of Variance

Notes

Output Created		27-NOV-2018 12:22:28
Comments		
Input	Data	C:\Users\c1571923\Desktop\GDM project\100 gdm a paper+median.sav
	Active Dataset	DataSet1
	Filter	<none>
	Weight	<none>
	Split File	Recorded in notes: Fetal Sex
	N of Rows in Working Data File	100
Missing Value Handling	Definition of Missing	User-defined missing values are treated as missing.
	Cases Used	Statistics are based on all cases with valid data for all variables in the model.
Syntax		UNIANOVA Telomere_percentage BY Groups_statistics WITH Caucasian_yes_no /METHOD=SSTYPE(3) /INTERCEPT=INCLUDE /EMMEANS=TABLES (Groups_statistics) WITH (Caucasian_yes_no=MEAN) COMPARE ADJ(LSD) /PRINT=DESCRIPTIVE /CRITERIA=ALPHA(.05) /DESIGN=Caucasian_yes_no Groups_statistics.
Resources	Processor Time	00:00:00.00
	Elapsed Time	00:00:00.00

Between-Subjects Factors

Recorded in notes: Fetal Sex			Value Label	N
Male	Groups_statistics	1.00	control	38
		2.00	gdm	10
		3.00	gdm+m	10
Female	Groups_statistics	1.00	control	31
		2.00	gdm	4
		3.00	gdm+m	7

Descriptive Statistics

Dependent Variable: Lower 5kb

Recorded in notes: Fetal Sex	Groups_statistics	Mean	Std. Deviation	N
Male	control	22.8147	12.21692	38
	gdm	32.5080	16.60049	10
	gdm+m	14.2850	7.11992	10
	Total	23.0153	13.32694	58
Female	control	22.7284	12.18061	31
	gdm	21.3700	11.02284	4
	gdm+m	31.2814	20.31744	7
	Total	24.0245	13.74082	42

Tests of Between-Subjects Effects

Dependent Variable: Lower 5kb

Recorded in notes: Fetal Sex	Source	Type III Sum of Squares	df	Mean Square
Male	Corrected Model	1987.182[a]	3	662.394
	Intercept	4232.795	1	4232.795
	Caucasian_yes_no	322.359	1	322.359
	Groups_statistics	1401.534	2	700.767
	Error	8136.430	54	150.675
	Total	40846.566	58	
	Corrected Total	10123.612	57	
Female	Corrected Model	521.697[b]	3	173.899
	Intercept	1095.714	1	1095.714
	Caucasian_yes_no	72.794	1	72.794
	Groups_statistics	416.823	2	208.412
	Error	7219.523	38	189.987
	Total	31982.685	42	
	Corrected Total	7741.220	41	

Tests of Between-Subjects Effects

Dependent Variable: Lower 5kb

Recorded in notes: Fetal Sex	Source	F	Sig.
Male	Corrected Model	4.396	.008
	Intercept	28.092	.000
	Caucasian_yes_no	2.139	.149
	Groups_statistics	4.651	.014
	Error		
	Total		
	Corrected Total		
Female	Corrected Model	.915	.443
	Intercept	5.767	.021
	Caucasian_yes_no	.383	.540
	Groups_statistics	1.097	.344
	Error		
	Total		
	Corrected Total		

a. R Squared = .196 (Adjusted R Squared = .152)

b. R Squared = .067 (Adjusted R Squared = -.006)

Estimated Marginal Means

Groups_statistics

Estimates

Dependent Variable: Lower 5kb

Recorded in notes: Fetal Sex	Groups_statistics	Mean	Std. Error	95% Confidence Interval	
				Lower Bound	Upper Bound
Male	control	22.586[a]	1.997	18.582	26.591
	gdm	32.242[a]	3.886	24.452	40.033
	gdm+m	15.419[a]	3.958	7.483	23.356
Female	control	22.721[b]	2.476	17.710	27.733
	gdm	21.800[b]	6.927	7.778	35.823
	gdm+m	31.066[b]	5.221	20.496	41.636

a. Covariates appearing in the model are evaluated at the following values: Caucasian_yes_no = 1.1379.

b. Covariates appearing in the model are evaluated at the following values: Caucasian_yes_no = 1.0952.

Pairwise Comparisons

Dependent Variable: Lower 5kb

Recorded in notes: Fetal Sex	(I) Groups_statistics	(J) Groups_statistics	Mean Difference (I-J)	Std. Error
Male	control	gdm	-9.656*	4.363
		gdm+m	7.167	4.461
	gdm	control	9.656*	4.363
		gdm+m	16.823*	5.572
	gdm+m	control	-7.167	4.461
		gdm	-16.823*	5.572
Female	control	gdm	.921	7.357
		gdm+m	-8.345	5.778
	gdm	control	-.921	7.357
		gdm+m	-9.266	8.702
	gdm+m	control	8.345	5.778
		gdm	9.266	8.702

Pairwise Comparisons

Dependent Variable: Lower 5kb

Recorded in notes: Fetal Sex	(I) Groups_statistics	(J) Groups_statistics	Sig.[b]	95% Confidence ...[b]
				Lower Bound
Male	control	gdm	.031	-18.403
		gdm+m	.114	-1.777
	gdm	control	.031	.910
		gdm+m	.004	5.651
	gdm+m	control	.114	-16.110
		gdm	.004	-27.995
Female	control	gdm	.901	-13.972
		gdm+m	.157	-20.041
	gdm	control	.901	-15.815
		gdm+m	.294	-26.882
	gdm+m	control	.157	-3.352
		gdm	.294	-8.350

Pairwise Comparisons

Dependent Variable: Lower 5kb

Recorded in notes: Fetal Sex	(I) Groups_statistics	(J) Groups_statistics	95% Confidence Interval for ...[b] Upper Bound
Male	control	gdm	-.910
		gdm+m	16.110
	gdm	control	18.403
		gdm+m	27.995
	gdm+m	control	1.777
		gdm	-5.651
Female	control	gdm	15.815
		gdm+m	3.352
	gdm	control	13.972
		gdm+m	8.350
	gdm+m	control	20.041
		gdm	26.882

Based on estimated marginal means

*. The mean difference is significant at the .05 level.

b. Adjustment for multiple comparisons: Least Significant Difference (equivalent to no adjustments).

Univariate Tests

Dependent Variable: Lower 5kb

Recorded in notes: Fetal Sex		Sum of Squares	df	Mean Square	F	Sig.
Male	Contrast	1401.534	2	700.767	4.651	.014
	Error	8136.430	54	150.675		
Female	Contrast	416.823	2	208.412	1.097	.344
	Error	7219.523	38	189.987		

The F tests the effect of Groups_statistics. This test is based on the linearly independent pairwise comparisons among the estimated marginal means.
